# Plasma microRNA-320c as a Potential Biomarker for the Severity of Knee Osteoarthritis and Regulates cAMP Responsive Element Binding Protein 5 (CREB5) in Chondrocytes

**DOI:** 10.1155/2024/9936295

**Published:** 2024-03-20

**Authors:** Rongwei Zhou, Like Zhao, Qian Wang, Yongjing Cheng, Miao Song, Cibo Huang

**Affiliations:** ^1^Department of Respiratory and Critical Care Medicine, School of Medicine, Shanghai Sixth People's Hospital Affiliated to Shanghai Jiao Tong University, Shanghai 200233, China; ^2^Department of Rheumatology, Beijing Hospital, National Center of Gerontology, Institute of Geriatric Medicine, Chinese Academy of Medical Sciences, Beijing 100730, China; ^3^Department of Emergency Medicine, Shanghai Eighth People's Hospital, Shanghai 200235, China

## Abstract

**Objective:**

Osteoarthritis (OA) is a commonly known prevalent joint disease, with limited therapeutic methods. This study aimed to investigate the expression of plasma microRNA-320c (miR-320c) in patients with knee OA and to explore the clinical value and potential mechanism of miR-320c in knee OA.

**Methods:**

Forty knee OA patients and 20 healthy controls were enrolled. The levels of plasma miR-320c and plasma inflammatory cytokines were measured by real-time PCR or ELISA. Correlations of Western Ontario and McMaster Universities Osteoarthritis Index (WOMAC) scores and cytokine levels with the miR-320c expression level were evaluated by Pearson correlation analysis. Then, a receiver operating characteristic (ROC) curve was drawn to analyse the diagnostic value of miR-320c in OA. Finally, the interaction of miR-320c and cAMP responsive element binding protein 5 (CREB5) was determined using a luciferase reporter assay, and the effect of CREB5 on the cAMP pathway was assessed.

**Results:**

The expression level of plasma miR-320c was significantly higher in OA patients than in healthy controls (*p*  < 0.05). The increased plasma miR-320c level was positively correlated with the WOMAC score (*r* = 0.796, *p*  < 0.001) and the plasma interleukin (IL)-1*β* (*r* = 0.814, *p*  < 0.001) and IL-6 (*r* = 0.695, *p*  < 0.001) levels in patients with OA. ROC curve analysis demonstrated the relatively high diagnostic accuracy of plasma miR-320c for OA. Furthermore, the luciferase reporter assay results showed that miR-320c regulates CREB5 expression by binding to the CREB5 3′-untranslated region. Moreover, suppression of CREB5 significantly reduced the expression levels of c-fos and c-jun.

**Conclusion:**

Our results indicate that plasma miR-320c may serve as a potential novel predictor of the severity of knee OA and that miR-320c may play an important role in the pathogenesis of OA through inhibiting the cAMP pathway by targeting CREB5.

## 1. Introduction

Osteoarthritis (OA) is the most common type of arthritis with an age-associated increase in both incidence and prevalence and affects tens of millions of people worldwide [1, 2]. It has become a major public health problem and has resulted a considerable socioeconomic burden [3]. As a dominant type of OA, knee OA is characterized by joint pain, stiffness, swelling, and decreased range of motion as well as snapping. Radiographic examination is not sensitive for early diagnosis. Currently, the molecular mechanisms involved in knee OA remain unclear, and there is no effective therapy to completely prevent the onset and progression of OA. Therefore, it is urgent to discover new effective and reliable biomarkers for knee OA.

MicroRNAs (miRNAs) are a subset of the large family of short (19–25 nucleotides) noncoding RNAs [4]. miRNAs can block the translation process and regulate gene expression posttranscriptionally by directly binding to complementary sequences in the 3′-untranslated regions (3′-UTRs) of target messenger RNAs (mRNAs) [5]. miRNAs are critical to cell physiology and development. They can modulate different biological processes, including cell differentiation, proliferation, apoptosis, and inflammation [6]. They have emerged as potentially useful biomarkers for risk assessment, diagnosis, and prognosis. Many miRNAs have been reported to play key roles in OA pathogenesis [7, 8]. Recent reports suggest that circulating miRNAs have great potential as diagnostic and prognostic biomarkers in connective tissue diseases, such as rheumatoid arthritis, systemic lupus erythematosus, and ankylosing diseases [9–12]. We previously reported that plasma miR-320c may be involved in the development of OA, as indicated by miRNA chip screening [13]. To date, few studies have addressed the mechanism of miR-320 in OA development [14–16]. The biological functions of miR-320c are still largely unknown, and its role in OA disease progression is even less understood.

OA is characterized by degeneration of cartilage. Accumulating evidence indicates that inflammation plays an important role in the pathophysiology of OA [17–19]. Inflammatory cytokines can control degeneration of the articular cartilage matrix [20, 21]. Many cytokines have been reported to be involved in OA, for example, tumor necrosis factor (TNF)-*α*, interleukin (IL)-1*β*, IL-4, IL-10, and IL-13. However, the interactions between miRNAs and cytokines in OA are not yet clear.

Hence, in the present study, we investigated the role of miR-320c in the progression of OA by analyzing its expression level and diagnostic value. Subsequently, correlation analysis between the miR-320c expression level, functional scores and cytokines expression level were performed. Then, analysis with bioinformatics software revealed that cyclic AMP (cAMP) responsive element binding protein 5 (CREB5) contains a complementary binding site for miR-320c. Further functional experiments were also carried out to explore the underlying mechanism of miR-320c and CREB5 in the pathogenesis of OA.

## 2. Materials and Methods

### 2.1. Patients and Sample Collection

A total of 40 patients with knee OA who received treatment at Beijing Hospital from Jan 2019 to May 2019 and 20 healthy control subjects with no history of autoimmune diseases, pulmonary diseases, or chronic diseases during the same period were enrolled. Healthy volunteers signed the informed consent form after recruitment through poster advertisement, and knee OA patients were enrolled after signing the informed consent form as inpatients. The patients with knee OA met the 1986 American College of Rheumatology revised classification criteria for knee OA [22]. Peripheral blood was collected and demographic characteristics were obtained from all subjects. Knee radiographs of all subjects were obtained and evaluated. The radiographic disease severity in knee OA patients was evaluated according to the Kellgren–Lawrence (KL) system, and the patients were divided into a moderate OA group (KL grade: I/II) and a severe OA group (KL grade: III/IV) [23]. The functional status of knee OA patients was evaluated using the Western Ontario and McMaster Universities Osteoarthritis Index (WOMAC) score [24]. A higher score on the WOMAC scale represents poorer function or greater pain, and the score is directly proportional to the severity of disease. Written informed consent was obtained from all participants, and the study was approved by the clinical Ethics Committee of Beijing Hospital (2018BJYYEC-141-01).

### 2.2. ELISA Test

Whole blood (5 ml) was placed into a sodium heparin tube, shake well, centrifuged at a speed of 3,000 rpm for 10 min at room temperature to separate plasma, and then stored in a −80°C refrigerator. Plasma levels of IL-1*β*, IL-6, IL-17, TNF-*α*, transforming growth factor (TGF)-*β*, and matrix metalloproteinase (MMP)-13 were measured using a Bio-Plex system following the manufacturer's instructions (Beijing Qualityard Biotechnology, Beijing, China). All samples were assayed in duplicate.

### 2.3. Cell Culture

The human chondrocyte articular (HC-a) cell line was provided by the Shanghai Chinese Academy of Sciences Health Science Research Center (Shanghai, China). HC-a cells were cultured in DMEM (Gibco, USA) supplemented with 10% fetal bovine serum (FBS; Gibco, USA) and 1% penicillin–streptomycin in a humidified atmosphere containing 5% CO_2_ at 37°C. Passage three chondrocytes were used in the following experiments, and all experiments were independently repeated three times in this study.

### 2.4. qRT–PCR

qRT–PCR was used to measure miR-320c expression in plasma and CREB5 expression in HC-a cells. Total RNA was extracted using TRIzol reagent (Invitrogen, USA) according to the manufacturer's protocol. The concentration and purity of RNA were determined using a Nanodrop2000 spectrophotometer (Thermo Fisher, USA). cDNA was synthesized using a PrimeScriptTM RT Reagent Kit (TaKaRa, Japan) according to the recommendations of the manufacturer. A TransStart SYBR Green qPCR SuperMix Kit (TransGen, China) and a TaqMan miRNA Reverse Transcription Kit (Thermo Fisher, USA) were used to perform RT–PCR analysis of CREB5 and miR-320c. Transcripts of either U6 (for miRNAs) or GAPDH (for mRNAs) served as the internal reference. The sequences of the primers used are listed in Table 1.

### 2.5. Transient Transfection

The miR-320c mimic, miR-320c mimic control, miR-320c inhibitor, and miR-320c inhibitor control were purchased from GenePharma (Shanghai, China). Small interfering RNA (siRNA) targeting CREB5 and negative control siRNA were purchased from Beijing Qualityard Biotechnology. HC-a cells (2.5 x 10^5^ cells/well) were maintained in 6-well plates to a confluence of 60%–70%. Twenty-four hours after seeding the cells were transfected with miR-320c mimic or inhibitor or siRNA utilizing Lipofectamine 3,000 (Invitrogen, USA) in serum-free medium. After 6 hr the culture medium was changed to regular medium containing antibiotics and serum. Forty-eight hours after transfection, the cells were collected for further experiments.

### 2.6. Plasmid Construction and Dual Luciferase Reporter Assay

The miR-320c targets were predicted using the TargetScan (http://www.targetscan.org/) database. A CREB5-3′UTR sequence containing the miR-320c binding site and another sequence lacking this site were synthesized using PCR amplification. The wild-type (Wt) and mutant (Mut) 3′-UTR sequences of CREB5 were inserted separately into the pmir-GLO luciferase reporter vector (Promega, USA). The recombinant plasmids were named pmir-GLO CREB5-3′-UTR Wt and pmir-GLO CREB5-3′-UTR Mut. To evaluate the effects of CREB5 on the cAMP pathway, the c-jun and c-fos promoter sequences were synthesized using PCR amplification and inserted separately into the pGL4.10 luciferase reporter vector (Promega, USA). The recombinant plasmids were named pGL4.10-fos promoter and pGL4.10-jun promoter. The primer sequences are listed in Table 1.

Then, we purchased stealth RNAi siRNA targeting CREB5 (Ruibo Biotechnology Co., Ltd., Guangzhou, China) and negative control siRNA. For specificity of the silencing effect, two siRNAs were used. The plasmids and miRNAs or siRNAs were cotransfected into HC-a cells. All luciferase activity was measured 24 hr post transfection by using a Dual-Luciferase Reporter Assay System (Promega, USA) according to the manufacturer's instructions. The firefly luciferase reading was normalized to the Renilla luciferase reading. Each experiment was repeated at least three times.

### 2.7. Western Blotting

Total protein was extracted 48 hr post transfection using RIPA Lysis Buffer (Pierce, USA) according to the manufacturer's instructions. The protein concentration was determined by using a Bicinchoninic Acid Assay Kit (Thermo Fisher, USA). Equal amounts of protein (20 *µ*g) were separated by SDS–PAGE (10%) and were then transferred to a polyvinylidene difluoride (PVDF) membrane (Thermo Fisher, USA). The membrane was blocked at room temperature for 1 hr using 5% nonfat milk and incubated overnight at 4°C with primary antibodies against CREB5 (1 : 1,000, Proteintech, USA), c-jun (1 : 1,000, Abcam, UK), c-fos (1 : 1,000, Abcam, UK), and GAPDH (1 : 5,000, Santa Cruz, USA). After washing three times with PBS, the membrane was incubated with a horseradish peroxidase (HRP)-conjugated anti-rabbit or anti-mouse secondary antibody (1 : 10,000, Santa Cruz, USA) at room temperature for 1 hr. *β*-Actin was used as the internal control.

### 2.8. Statistical Analysis

Statistical analysis was performed using SPSS 23.0 (SPSS, USA) and GraphPad Prism (Version 7.0, USA) software. The data are presented as the mean ± standard deviation (SD) values. The normality of the data was tested by the Shapiro–Wilk test in SPSS software. Two-tailed Student's *t* test or one-way analysis of variance (ANOVA) was used to analyze the significance of differences between and among groups, respectively. The Pearson correlation coefficient was used to assess the correlations between the miR-320c level and different factors. Receiver operating characteristic (ROC) curves were used to evaluate the sensitivity and specificity of miR-320c for discriminating patients with knee OA. A *p* value < 0.05 was considered statistically significant.

## 3. Results

### 3.1. Clinical Characteristics of the Study Subjects

Forty knee OA patients and 20 age-matched healthy controls were recruited. The knee OA patients were divided into a moderate OA group (*n* = 20) and a severe OA group (*n* = 20) according to the KL grade. The mean ages in the healthy control group, moderate OA group, and severe OA group were 58.15 ± 9.84, 61.15 ± 11.64, and 63.25 ± 9.73 years (*p* > 0.05), respectively. There was no significant gender difference among the healthy control group (male: 55% and female: 45%), moderate OA group (male: 30% and female: 70%), and severe OA group (male: 40% and female: 60%; Pearson *χ*2 = 2.606, *p*=0.272).

### 3.2. Correlation of the Plasma miR-320c Level with the KL Grade and WOMAC Score

**Figure 1:**
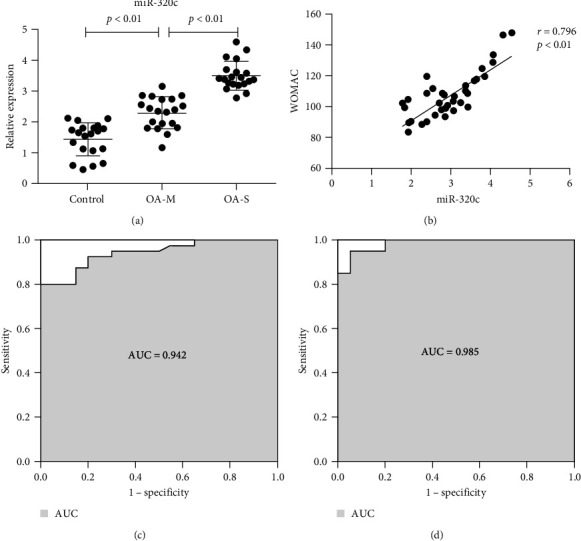
Expression level and diagnostic value of plasma miR-320c in osteoarthritis: (a) the expression levels of plasma miR-320c in the OA groups and control group by qRT-PCR (normalized to U6); (b) correlation of the plasma miR-320c level with the Western Ontario and McMaster Universities Osteoarthritis Index (WOMAC) score; (c) receiver operating characteristic (ROC) curve for plasma miR-320c in OA, and the area under the curve (AUC) was 0.942; and (d) ROC curve for plasma miR-320c between moderate and severe OA, and the AUC was 0.985. OA-M: moderate osteoarthritis group and OA-S: severe osteoarthritis group.

The expression level of plasma miR-320c was significantly higher in both the moderate OA group (2.30 ± 0.51) and the severe OA group (3.51 ± 0.47) than in the control group (1.44 ± 0.54; *p* < 0.01; Figure 1(a)). As the radiographic stage increased, the expression level of miR-320c increased (Figure 1(a)). Then, we performed logistic regression analysis and found that the miR-320c expression level was positively associated with the radiological severity (*r* = 0.865, *p*=0.007). Moreover, the expression level of plasma miR-320c was positively correlated with the WOMAC score in knee OA patients (*r* = 0.796, *p* < 0.01; Figure 1(b)).

### 3.3. ROC Curve of Plasma miR-320c in OA

To improve the potential diagnostic efficiency of plasma miR-320c in OA patients, ROC curve analysis of miR-320c was performed to discriminate patients with OA from healthy controls. The AUC was 0.942 (95% CI: 0.888−0.996; *p* < 0.01). At the optimal cutoff value of 2.13, the diagnostic sensitivity and specificity of miR-320c were 80.0% and 95.0%, respectively (Figure 1(c)). Interestingly, we found through ROC curve analysis between moderate and severe OA that miR-320c can assess the severity of OA, with an AUC of 0.985 (95% CI: 0.958–1.000; *p*  < 0.01). At the optimal critical value of 2.905, the sensitivity and specificity of miR-320c in diagnosing the severity of OA were 95.0% and 95.0%, respectively (Figure 1(d)).

### 3.4. The Plasma miR-320c Level Was Positively Correlated with IL-1*β* and IL-6 Levels

**Figure 2:**
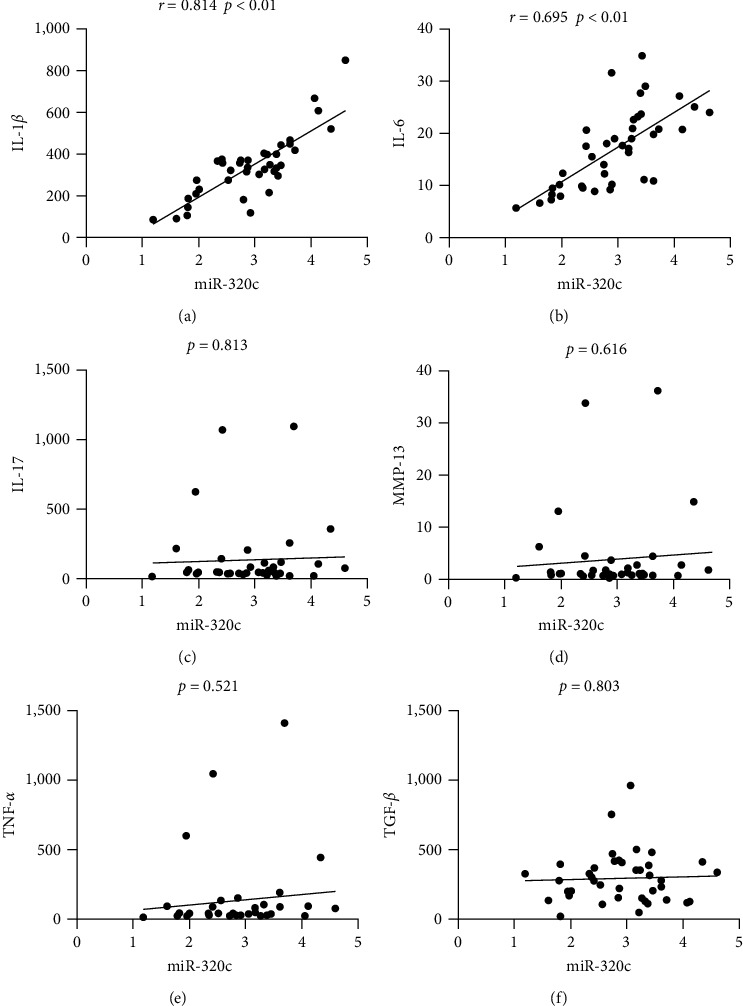
Correlations of the plasma miR-320c level and cytokine expression levels in osteoarthritis patients. Pearson correlation analysis was performed for: (a) IL-1*β*, (b) IL-6, (c) IL-17, (d) MMP-13, (e) TNF-*α*, and (f) TGF-*β*. The unit of all cytokine levels is pg/mL.

The expression levels of cytokines (IL-1*β*, IL-6, IL-17, and TNF-*α*), MMP-13, and TGF-*β* in the OA groups were also measured by ELISA. As shown in Figure 2, the plasma miR-320c expression level was positively correlated with the expression levels of IL-1*β* and IL-6 (*r* = 0.814, *p* < 0.01 and *r* = 0.695, *p* < 0.01; respectively). miR-320 c was not correlated with IL-17, MMP-13, TNF-*α*, or TGF-*β* (all *p* > 0.05).

### 3.5. MiR−320 c Negatively Regulated CREB5 Expression

**Figure 3:**
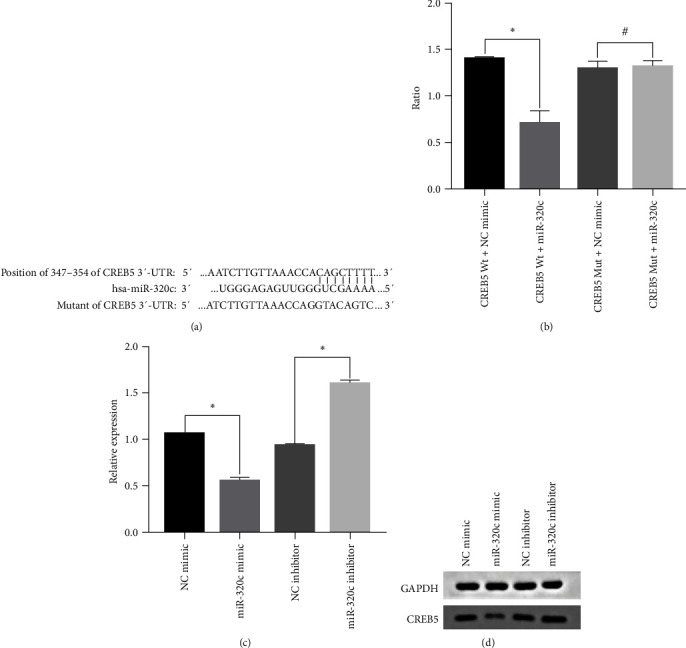
miR-320c negatively regulated CREB5 expression by targeting the CREB5 3′-untranslated region (UTR): (a) binding site for miR-320c in the CREB5 3′-UTR; (b) relative luciferase activity of CREB5 in HC-a cells (normalized to the Renilla luciferase); (c) CREB5 mRNA expression level, as determined by real-time PCR (normalized to GAPDH); and (d) CREB5 protein expression level, as determined by western blot analysis. Two-tailed student's *t* test was used to compare between groups.  ^*∗*^*p* < 0.01, ^#^*p* > 0.05. Wt: wild-type. Mut: mutant. Ratio: firefly luciferase activity/Renilla luciferase activity.

Bioinformatics prediction was performed to identify target genes of miR-320c, and the putative binding site for miR-320c in the 3′-UTR of CREB5 was predicted (Figure 3(a)). The reporter vectors containing the CREB5 3′-UTR Wt/Mut sequences and the miR-320c mimic/control were cotransfected into HC-a cells.

The luciferase reporter assay results showed that overexpression of miR-320c (miR-320c + CREB5 3′-UTR Wt group) significantly decreased the relative luciferase activity in cells in the miRNA NC + CREB5 3′-UTR Wt group (*p* < 0.01, Figure 3(b)). However, no significant difference in luciferase activity was observed between the miRNA NC + CREB5 3′-UTR Mut group and the miRNA NC + CREB5 3′-UTR Mut group (Figure 3(b), *p* > 0.05). In addition, the relative luciferase activity was almost the same in the miRNA NC + CREB5 3′-UTR Mut group and the miRNA NC + CREB5 3′-UTR Wt group.

Furthermore, the miR-320c mimic, mimic control, miR-320c inhibitor, and inhibitor control were transfected separately into HC-a cells. RT–PCR and western blotting were performed to measure the mRNA and protein expression levels of CREB5. The results showed that both the mRNA and protein expression levels of CREB5 were significantly decreased in the miR-320c mimic group and dramatically increased in the miR-320c inhibitor group (Figures 3(c) and 3(d); *p* < 0.01). Collectively, these results indicate that miR-320c negatively regulates the expression of CREB5 by directly binding to the CREB5 3′-UTR.

### 3.6. Downregulation of CREB5 Reduced c-fos and c-jun Expression in HC-a Cells

**Figure 4:**
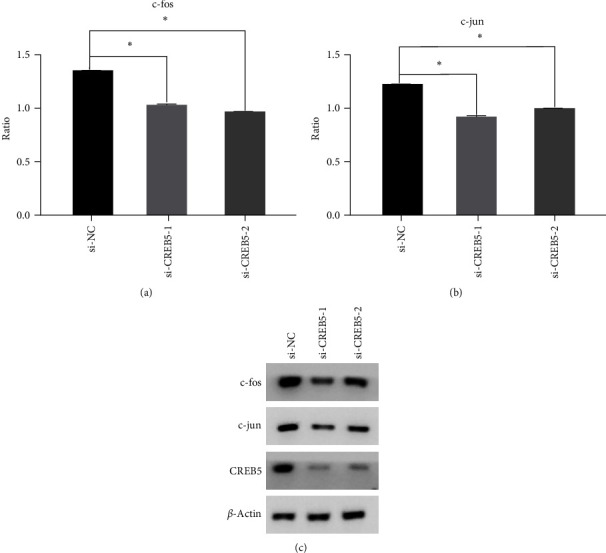
Downregulation of miR-320c reduced c-fos and c-jun expression: (a) relative luciferase activity of c-fos in HC-a cells. The ratio of firefly luciferase/Renilla luciferase was calculated; (b) relative luciferase activity of c-jun in HC-a cells. The ratio of firefly luciferase/Renilla luciferase was calculated; and (c) protein expression levels of c-fos, c-jun, and CREB5 (normalized to *β*-actin). si-NC: negative control, and si-CREB5-1 and si-CREB5-2: two different sites for CREB5. One-way analysis of variance (ANOVA) was used to compare luciferase activity between groups.  ^*∗*^*p* < 0.01.

The effects of CREB5 on the expression of c-fos and c-jun in HC-a cells were evaluated. The plasmids (pGL4.10-fos promoter/pGL4.10-jun promoter) and si-CREB5/si-Control were cotransfected into HC-a cells. The luciferase reporter assay and western blot analysis results demonstrated that suppression of CREB5 expression by siRNA significantly reduced the expression levels of c-fos and c-jun (Figure 4). These data indicate that CREB5 suppresses the activation of the cAMP pathway.

## 4. Discussion

The results of our study showed that plasma miR-320c expression is high in OA patients and may serve as a promising biomarker to predict the severity of knee OA. Furthermore, we revealed that a high expression level of miR-320c was positively associated with a high KL grade, a high WOMAC score, and high inflammatory cytokine (IL-1*β* and IL-6) levels. Moreover, we found that miR-320c can negatively regulate the expression of CREB5 and that knockdown of CREB5 can suppress the expression of c-fos and c-jun.

miRNAs have been shown to be present in several types of body fluids, such as serum and plasma [25]. Circulating miRNAs are sensitive as markers, easily detectable and highly stable; thus, they are used as noninvasive diagnostic biomarkers and novel therapeutic targets in various diseases [26–28]. Recent evidence has demonstrated that multiple miRNAs are differentially expressed in OA and are considered potential diagnostic biomarkers and therapeutic targets in OA [29, 30]. The current results revealed that the expression level of plasma miR-320c was significantly increased in OA patients. miR-320 has been reported to be related to suppression of cell proliferation and apoptosis and may act as a biomarker for prognosis and the therapeutic response in cancer [31–33]. In this study, ROC curve analysis demonstrated the relatively high diagnostic accuracy of plasma miR-320c for OA patients, with relatively high sensitivity and specificity. Furthermore, the correlations of plasma miR-320c expression with imaging severity and physiological severity were analyzed. Imaging severity and physiological severity are commonly evaluated by using the KL grading scale and WOMAC score for OA patients. Our results revealed that plasma miR-320c expression was increased with increasing radiographic stage and was positively correlated with the WOMAC score. These results suggest that plasma miR-320c can be used as a potential monitoring indicator for the radiographical severity and physiological severity of knee OA.

Cytokines are the main players in many inflammatory conditions, including OA [21, 34, 35]. The expression of IL-1*β* and IL-6 was increased in OA patients in this study, consistent with previous findings. IL-1*β* and IL-6 are important proinflammatory and catabolic cytokines in OA. Previous studies have shown that IL-1*β* can increase the expression and activity of key enzymes in matrix degradation and decrease the synthesis of crucial extracellular cartilage matrix components [36]. Furthermore, IL-1*β* induces the production of a number of cytokines and chemokines that contribute to inflammation [37]. Studies have demonstrated that IL-6 can stimulate synoviocyte proliferation and osteoclast activation and is associated with increased levels of cartilage-degrading matrix metalloproteinase enzymes [38, 39]. Our results showed that the expression of miR-320c was positively correlated with that of IL-1*β* and IL-6, which reveals that the expression level of plasma miR-320c may reflect the inflammatory state in OA.

Additionally, the underlying mechanism of miR-320c in OA was investigated by using a luciferase assay system. Previous studies showed that miR-320c can target different genes, such as SP1 [33], PBX3 [31], E2F1 [40], and AKIP1 [41]. In our previous research, we conducted signal pathway clustering analysis using predicted miR-320c target genes and selected cAMP signal pathways based on enrichment levels [42]. CREB5 was selected as target gene through establishing regulatory network between miR-320c and the predicted target gene. CREB5 is a member of the cAMP response element binding protein family. As a second messenger, cAMP always plays a role by activating protein kinase A to promote the transcription and translation of the downstream genes c-jun and c-fos. It also participates in muscle contraction, neurotransmission, vision, differentiation, cell growth, and exocytosis [43]. Activation of the cAMP pathway in osteoblasts decreases the inhibition of osteoclastogenesis [44]. To date, studies investigating the effect of miR-320c on CREB5 and the cAMP pathway are limited. The present results confirmed that miR-320c can negatively regulate CREB5 expression by directly targeting the 3′-UTR of CREB5 in HC-a cells. Functional studies showed that knockdown of CREB5 decreased the c-jun and c-fos expression levels in HC-a cells. Indirectly, the results indicated that miR-320c is probably involved in the pathogenesis of OA by affecting the cAMP pathway.

At present, there are no specific biomarkers for disease activity in OA, and there is a lack of simple and feasible monitoring indicators that can reflect the severity of the disease. The application of imaging to evaluate the severity of diseases in clinical practice is far from sufficient, as X-rays are insensitive and delayed, and MRI is expensive. Our research findings suggest the feasibility of using serological indicators to evaluate the severity of OA, which can further guide precision treatment in clinical practice in the future.

However, there are still some limitations in our study. First, the number of participants enrolled in the study was limited. The diagnostic value of miR-320c in OA needs to be investigated in a large-scale study. Second, the mechanism of miR-320c and cAMP in the development of OA needs to be verified by in vitro experiments in cells and in vivo experiments in animals. Further studies are needed to confirm the effects of miR-320c on the cAMP pathway.

In conclusion, we investigated the clinical role of plasma miR-320c in OA and explored its underlying mechanism in the pathogenesis of OA. High miR-320c levels are related to disease severity and inflammation status, and miR-320c may be involved in the development of OA by influencing the cAMP pathway in chondrocytes.

## 5. Conclusions

This study demonstrated that plasma miR-320c may serve as a potential novel predictor of the severity of knee OA and that miR-320c may play an important role in the pathogenesis of OA through inhibiting the cAMP pathway by targeting CREB5.

## Figures and Tables

**Table 1:** Primer sequences used for vector construction and real-time PCR.

Gene	Primer sequence
miR-320c	F: AAAAGCTGGGTTGAGAGGGT
	R: ACCCTCTCAACCCAGCTTTT

CREB5	F: CCCTGCCCAACCCTACAATG
	R: GGACCTTGCATCCCCATGAT

CREB5 3′-UTR Wt	F: AGTCCAACCCTTTGCCTGAAA
	R: TGACACCACAGCACAAACTCA

CREB5 3′-UTR Mut	F: CTTGGGAAACGCTTTGGTGC
	R: TAGCTGACTGTACCTGGTTTAACA

c-jun promoter	F: GCAGCGGAGCATTACCTCAT
	R: TAGCCCATGATGTCACCCCA

c-fos promoter	F: GGGGACATGCGTCTTCGC
	R: CAAGGTGCTCCAGAGTGTGC

GAPDH	F: GGAGCGAGATCCCTCCAAAAT
	R: GGCTGTTGTCATACTTCTCATGG

U6	F: GCTTCGGCAGCACATATACTAAAAT
	R: CGCTTCACGAATTTGCGTGTCAT

F: forward; R: reverse; CREB5: cAMP responsive element binding protein 5; UTR: untranslated region; Wt: wild-type; and Mut: mutant.

## Data Availability

All data generated or analyzed during this study are included in this published article. The datasets used and/or analyzed during the current study are available from the corresponding author on reasonable request.
